# Identification of novel diagnostic biomarkers for thyroid carcinoma

**DOI:** 10.18632/oncotarget.22873

**Published:** 2017-12-04

**Authors:** Xiliang Wang, Qing Zhang, Zhiming Cai, Yifan Dai, Lisha Mou

**Affiliations:** ^1^ Shenzhen Xenotransplantation Medical Engineering Research and Development Center, Institute of Translational Medicine, Shenzhen Second People's Hospital, First Affiliated Hospital of Shenzhen University, Shenzhen 518035, China; ^2^ Department of Biochemistry in Zhongshan School of Medicine, Sun Yat-Sen University, Guangzhou 510080, China; ^3^ Jiangsu Key Laboratory of Xenotransplantation, Nanjing Medical University, Nanjing 210029, China

**Keywords:** thyroid carcinoma, bioinformatics, dysregulation network, biomarker

## Abstract

Thyroid carcinoma (THCA) is the most universal endocrine malignancy worldwide. Unfortunately, a limited number of large-scale analyses have been performed to identify biomarkers for THCA. Here, we conducted a meta-analysis using 505 THCA patients and 59 normal controls from The Cancer Genome Atlas. After identifying differentially expressed long non-coding RNA (lncRNA) and protein coding genes (PCG), we found vast difference in various lncRNA-PCG co-expressed pairs in THCA. A dysregulation network with scale-free topology was constructed. Four molecules (LA16c-380H5.2, RP11-203J24.8, MLF1 and SDC4) could potentially serve as diagnostic biomarkers of THCA with high sensitivity and specificity. We further represent a diagnostic panel with expression cutoff values. Our results demonstrate the potential application of those four molecules as novel independent biomarkers for THCA diagnosis.

## INTRODUCTION

Thyroid carcinoma (THCA) is the most widespread endocrine malignancy worldwide, with an incidence rate that increases by 4% every year [1, 2]. Tissue biopsy is the current gold standard for diagnostic tests for cancers; however, biopsy results are often inevitably subjective because of differing reporting methods among pathologists or limited diagnostic accuracy associated with sampling error [3]. For THCA, indeterminate (10-20%) and inadequate (10-15%) conclusions perplex clinicians and undermine the diagnostic value of biopsy procedures [4], and the use of core needle biopsy for the thyroid is currently limited by a lack of well-accepted diagnostic criteria [5]. Some THCAs are associated with aggressive clinical behavior or a poor prognosis [6]. Therefore, finding more sensitive and specific biomarkers to use for the early detection of THCA is undoubtedly of great significance. However, there is still no routine application of these markers in clinical practice. Progress in understanding the molecular basis of cancers using omics technologies has provided opportunities to develop novel tools to diagnose, predict cancers and evaluate treatment responses [7].

Long noncoding RNA (lncRNA) is unable to be translated into proteins, with transcript lengths of more than 200 nucleotides [8, 9]. LncRNA has emerged as a vital regulator in biological, developmental and pathological processes of tissues and diseases including various cancers through mechanisms such as chromatin reprogramming, *cis* regulation at enhancers and post-transcriptional regulation of mRNA processing [10, 11]. Cancer-related lncRNAs showed aberrant expression patterns in tissue- or cancer type-specific manners, suggesting their potentials as novel independent and promising biomarkers for cancer diagnosis or prognosis [12–16]. Recently, a very large-scale data analysis found 1289 THCA-associated lncRNAs, just after renal clear cell carcinoma (1429 associated lncRNAs), in 27 types of cancers [10]. However, a few lncRNAs were declared to be implicated in the development and progression of THCA. For instance, lncRNAs BANCR and PVT1 were overexpressed, and NAMA and PTCSC3 were down-regulated in THCA patients [17–20]. To date, limited knowledge is known about the diagnostic or prognostic values of lncRNA in THCA [21]. In this study, next generation sequencing (NGS) datasets from The Cancer Genome Atlas (TCGA) [22] were used to identify two lncRNAs (LA16c-380H5.2 and RP11-203J24.8) and two protein coding genes (PCGs) (MLF1 and SDC4) as potential diagnostic biomarkers with high sensitivity and specificity.

## RESULTS

### Transcriptome expression profiles in THCA and normal samples

**Figure 1:**
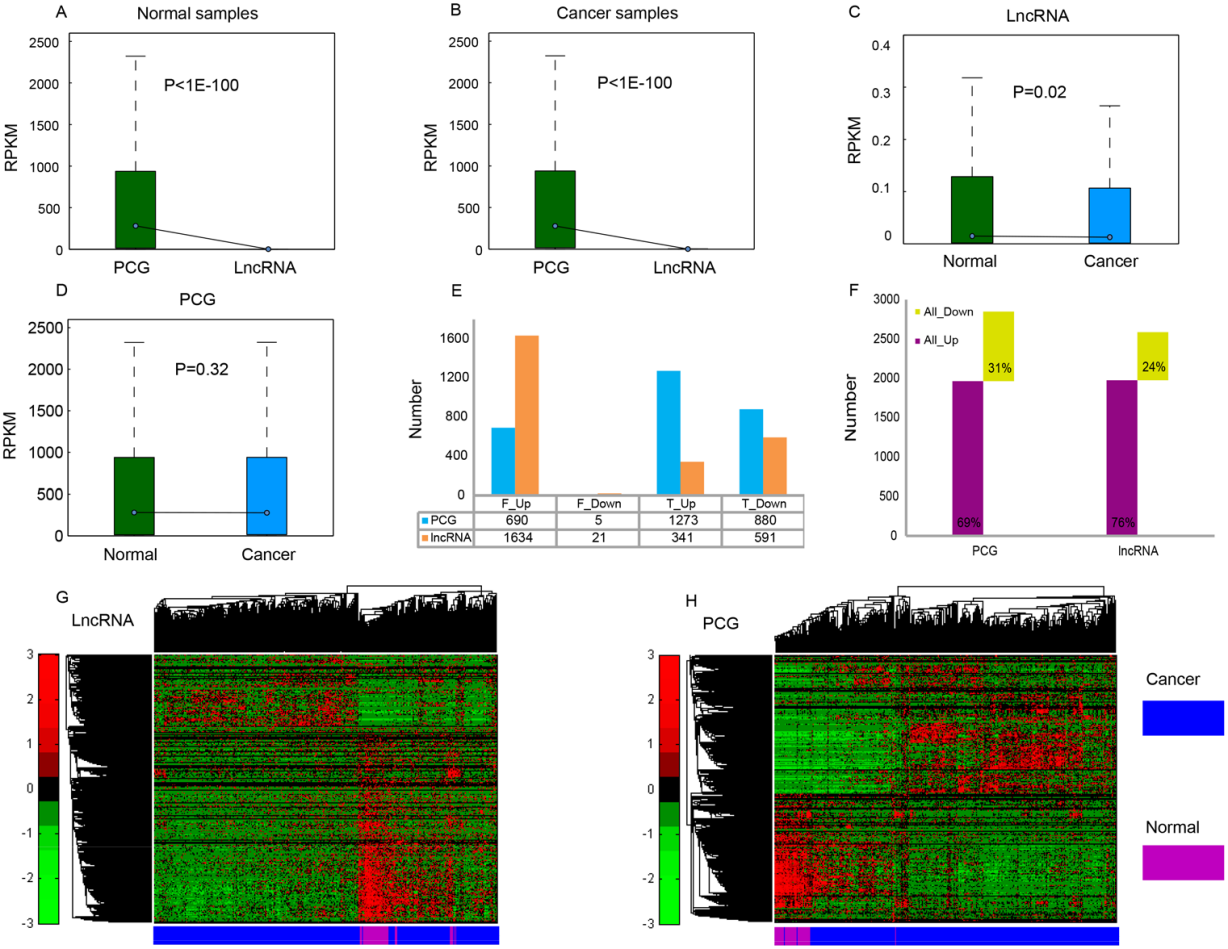
Expression profiles of PCG and lncRNA in cancer and normal samples Average expression levels (RPKM) of PCG and lncRNA in normal samples **(A)** and THCA samples **(B)**. RPKM comparison of lncRNA **(C)** and PCG **(D)** between normal samples and cancer samples. **(E)** The number of up- and down-regulated PCG and lncRNA identified through Fisher’s exact test and Student's t test, respectively. F_Up/F_Down: up- or down-regulated PCG or lncRNA identified through Fisher’s exact test, T_Up/T_Down: up- or down-regulated PCG or lncRNA identified through Student's t test. **(F)** Total number of differentially expressed PCG and lncRNA. Unsupervised hierarchical clustering using expression profiles of lncRNA **(G)** and PCG **(H)** revealed distinct separation of cancer samples from normal samples.

We compared expression levels of PCG and lncRNA between 505 cancer and 59 normal samples (sample list in Supplementary Table 1). LncRNA was expressed at evidently lower levels than PCG in both cancer and normal samples (P < 1E-100), showing a low abundance of lncRNA in cells (Figure 1A and 1B), which is consistent with previous studies [23, 24]. When compared with normal samples, the expression perturbation observed for lncRNA was more significant in THCA (P = 0.02, Figure 1C), while PCG variance was considerably weaker in cancer samples (P = 0.32, Figure 1D), partly because that the specific expression of lncRNA is a vital regulator in gene expression.

To identify differentially expressed PCGs and lncRNAs in THCA and normal thyroid glands, we applied a binary statistical analysis based on the number of zero-values in the expression level (read count = 0). We identified 690 up-regulated and five down-regulated PCGs, and 1634 up-regulated and 21 down-regulated lncRNAs with |fold change (FC)| > 2 and FDR < 0.01 by Fisher’s exact test; as well as 1273 up-regulated and 880 down-regulated PCGs, and 341 up-regulated and 591 down-regulated lncRNAs with |FC| > 2 and FDR < 0.05 by Student's t test (Figure 1E). In total, we found that 2848 PCGs and 2587 lncRNAs were differentially expressed between THCA and normal tissue (Supplementary Table 2). Among them, 1963 PCGs and 1975 lncRNAs were up-regulated, while 885 PCGs and 612 lncRNAs were down-regulated (Figure 1F). Up-regulated features were found to be much more common than down-regulated features (> 2 times), which is similar to the expression patterns in two other reports about THCA [4, 25]; these results likely indicate that the demands of cancer cells for quick proliferation, tissue invasion and metastasis are met. A hierarchical cluster analysis of differentially expressed lncRNA (Figure 1G) and PCG (Figure 1H) showed that THCA patients were well discriminated from normal individuals according to their expression levels, indicating the possibility of selecting a group of features for THCA diagnosis.

Pathway analysis of differentially expressed PCGs indicated that 34 pathways were enriched significantly (P < 0.05, Supplementary Table 3). Many of these pathways were linked to cancer, such as “pathways in cancer” (P = 0.028), “cytokine-cytokine receptor interaction” (P = 8.54E-6), which was in accordance with an early research [2], “PPAR signaling pathway” (P = 0.022), which was particularly associated with THCA [26, 27], and “PI3K-Akt signaling pathway” (P = 1.63E-4), which was one of the most important molecular mechanisms identified in the carcinogenesis of THCA [28].

### Dysregulated network of differentially expressed features

To explore the dysregulation of differentially expressed lncRNAs involved in THCA tumorigenesis and development, we calculated Pearson’s correlation coefficient (PCC) by examining the paired lncRNA and PCG expression profiles. In normal samples, 1085 lncRNAs were identified to have 8393 target PCGs with 193424 co-expressed pairs using all PCGs in the Ensembl reference (“AllPCG” for short). Among them, 1083 lncRNAs were positively correlated with 8154 PCGs with 190880 co-expressed pairs, while only 130 lncRNAs were negatively correlated with 1260 PCGs with 2544 co-expressed pairs (Table 1). When using those differentially expressed PCGs (“DiffPCG” for short), 795 lncRNAs were found to have 1270 target PCGs with 24752 co-expressed pairs. Among those, 793 lncRNAs were positively correlated with 1263 PCGs with 24668 pairs, while only 24 lncRNAs were negatively correlated with 47 PCGs with 84 pairs (Table 1). As for those differentially expressed PCGs that were also cancer genes (cancer genes were cited from the report of *Mathias Uhlén et al.* [29]; “CancerG” for short, Supplementary Figure 1), 282 lncRNAs had 37 target PCGs with 579 co-expressed pairs. All of the 282 lncRNAs were positively correlated with 37 PCGs with 578 pairs, while just one lncRNA was negatively correlated with one PCG with one pair (Table 1). Although there were positive and negative co-expression pairs of lncRNA-PCG in normal samples, the former was far more common than the latter.

**Table 1:** Number of differentially expressed lncRNA-PCG co-expression pairs

		Co-expression pairs			Positive pairs			Negative pairs		
		Pairs	lncRNA	PCG	Pairs	lncRNA	PCG	Pairs	lncRNA	PCG
AllPCG	Normal	193424	1085	8393	190880	1083	8154	2544	130	1263
	Cancer	8042	558	2091	8042	558	2091	0	0	0
	Total	199146	1317	9238	196602	1315	8999	2544	130	1263
DiffPCG	Normal	24752	795	1270	24668	793	1263	84	24	47
	Cancer	2131	436	633	2131	436	633	0	0	0
	Total	26455	1006	1568	26371	1004	1561	84	24	47
CancerG	Normal	579	282	37	578	282	37	1	1	1
	Cancer	36	32	16	36	32	16	0	0	0
	Total	604	296	42	603	296	42	1	1	1

Co-expressed pairs were defined with cutoff of |PCC| ≥ 0.7 and P < 0.001.

3th-5th column: number of co-expressed pairs between PCGs and differentially expressed lncRNAs.

6th-8th column: number of positively co-expressed pairs between PCGs and differentially expressed lncRNAs.

9th-11th column: number of negatively co-expressed pairs between PCGs and differentially expressed lncRNAs.

3th-4th row: number of co-expressed pairs between all PCGs in the Ensembl reference (“AllPCG” for short) and differentially expressed lncRNAs in normal (3th row) or THCA samples (4th row).

6th-7th row: number of co-expressed pairs between differentially expressed PCGs (“DiffPCG” for short) and differentially expressed lncRNAs in normal (6th row) or THCA samples (7th row).

9th-10th row: number of co-expressed pairs between those differentially expressed PCGs that were also cancer genes (“CancerG” for short) and differentially expressed lncRNAs in normal (9th row) or THCA samples (10th row).

In THCA patients, 558 lncRNAs had 2091 targets with 8042 co-expressed pairs in the “AllPCG” level. 436 lncRNAs had 633 targets with 2131 co-expressed pairs in the “DiffPCG” level. When using “CancerG”, 32 lncRNAs had 16 targets with 36 co-expressed pairs (Table 1). It was astounding that all of these lncRNAs were positively correlated with PCGs in THCA samples without negative regulation, showing that PCGs followed the same trends as lncRNAs (Figure 1F). Moreover, the number of co-expressed pairs in THCA patients was significantly less than in normal samples, only approximately 4.16% (8042/193424) in the “AllPCG” level, 8.61% (2131/24752) in the “DiffPCG” level, and 6.22% (36/579) in the “CancerG” level (Table 1), illustrating a massive loss in regulation of lncRNAs to PCGs in THCA tumorigenesis or development. This agreed with the idea that most characterized lncRNAs display deregulated expression in cancers, suggesting they may play oncogenic or tumor suppressive functions [30].

Four types of dysregulated pairs among lncRNA-PCG co-expressed pairs are defined in Table 2. There were 196826, 26027, and 593 dysregulated pairs, accounting for 98.84% (out of 199146), 98.38% (out of 26455), and 98.18% (out of 604) in “AllPCG”, “DiffPCG”, and “CancerG” levels, respectively (Table 1 and Table 2). In particular, in the “CancerG” level, nearly 100% of the lncRNAs (295/296) and PCGs (42/42) showed dysregulation (Table 2). It is no surprise that the overwhelming majority of the population was Type I; there were no Type III and Type IV dysregulated pairs (Table 2). Those lost (Type I) and gained (Type II) dysregulated pairs may be one important reason for the aberrance of THCA. These results showed dramatic turbulence in the regulation roles of lncRNA to PCG in THCA, supporting the findings that lncRNAs are frequently dysregulated in various tumors [31–33].

**Table 2:** Four types of dysregulated pairs among co-expressed pairs of lncRNA-PCG

Type	Normal	Cancer	AllPCG			DiffPCG			CancerG		
			lncRNA	PCG	Pairs	lncRNA	PCG	Pairs	lncRNA	PCG	Pairs
I	Yes	No	1060	8344	191104	764	1237	24324	281	36	568
II	No	Yes	490	1634	5722	371	549	1703	23	14	25
III	Positive	Negative	0	0	0	0	0	0	0	0	0
IV	Negative	Positive	0	0	0	0	0	0	0	0	0
Total			1298	9202	196826	985	1546	26027	295	42	593

I: co-expressed pairs appeared in normal samples, while disappeared in THCA. II: co-expressed pairs disappeared in normal samples, while appeared in THCA. III: pairs positively co-expressed in normal samples, while negatively co-expressed in THCA. IV: pairs negatively co-expressed in normal samples, while positively co-expressed in THCA.

AllPCG: all protein coding genes (PCGs) in the Ensembl reference. DiffPCG: differentially expressed PCGs. CancerG: differentially expressed PCGs that were also cancer genes.

The number of lncRNA in this table is the number of differentially expressed lncRNAs between normal and THCA samples.

**Figure 2:**
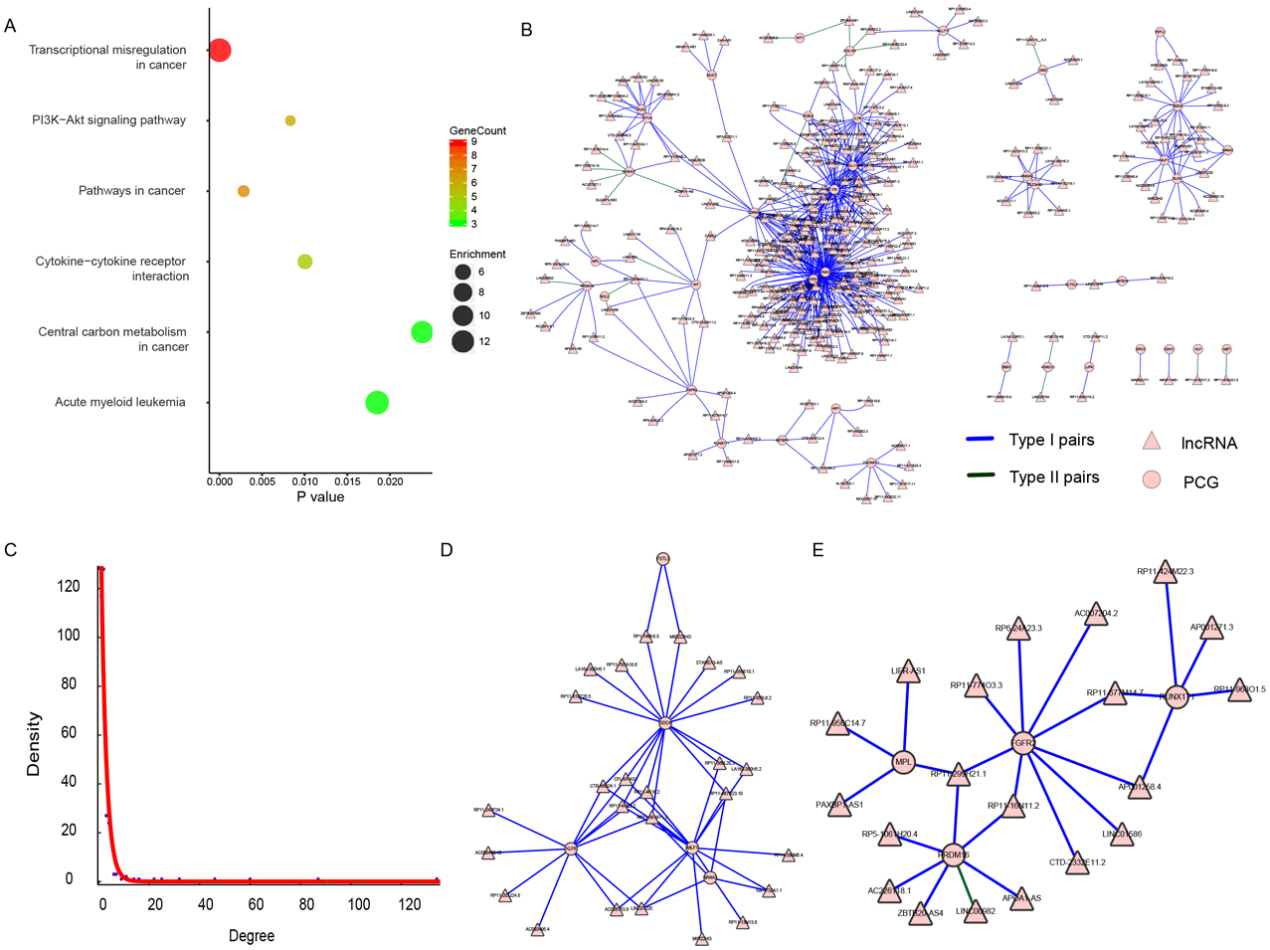
The lncRNA-PCG dysregulation network of the 42 PCGs **(A)** Results of KEGG pathway enrichment, showing P value, gene count, and fold enrichment in each pathway. **(B)** The dysregulation network of the 42 PCGs. The lncRNA was indicated to triangle and PCG was indicated to circle. The color of blue represents Type I (disappearance) pairs and green represents Type II (appearance) pairs. **(C)** The exponential distribution of degree of each node in (B). **(D)** and **(E)** were two sub-modules identified from (B).

Both in Table 1 and Table 2, there was an intriguing phenomenon where the number of lncRNAs was much lower than that of PCG in both “AllPCG” and “DiffPCG” levels, which was concordant with the idea that lncRNA could target multiple, even hundreds of genes in the human genome [34, 35]. This is just the opposite in the “CancerG” level where one PCG was influenced by several lncRNAs, suggesting key roles of those 42 cancer PCGs in THCA (42 PCGs are listed in Supplementary Table 4). For example, ENSG00000105976 (c-Met) is an oncogene protein with tyrosine kinase activity, and its abnormal activation has been detected in various cancers, including THCA [36, 37]. Another example is that ENSG00000066468 (FGFR-2), which has been implicated in the onset of THCA, was reduced in THCA [38, 39]. In agreement with the above reports, c-Met was aberrantly up-regulated (P < 2.2E-16, FC = 5.99), and FGFR-2 was aberrantly down-regulated (P < 2.2E-16, FC = 2.3) in our study (Supplementary Figure 2A and 2B). Pathway analysis of those 42 PCGs showed that “Transcriptional misregulation in cancer” was the most enriched pathway (P = 1.11E-7), followed by “Pathways in cancer” (P = 2.83E-3), “PI3K-Akt signaling pathway” (P = 8.32E-3), “Cytokine-cytokine receptor interaction” (P = 0.01), “Acute myeloid leukemia” (P = 0.019), and “Central carbon metabolism in cancer” (P = 0.024) (Figure 2A). All of those six pathways are associated with cancer, these key pathways are regulated by lncRNA in THCA.

Therefore, we focused on the co-expressed network based on dysregulation pairs of those 42 PCGs and 295 regulating lncRNAs (Table 2). There were 337 (42 and 295) nodes and 593 edges in the network (Figure 2B), which had a scale-free topology with degree distribution following a power law (Figure 2C). Such a scale-free network has been found in many different organizational levels, ranging from genetics to protein interaction and protein domains [40, 41]. A distinguishing feature of such a scale-free network is the existence of a few highly connected nodes [42]. There were 117 (72.7%) nodes of a degree lower than three, while 23 (6.8%) nodes had degrees of six or above. To test the existence of separable functional units, we tried to mine feasible sub-modules. Two sub-networks were finally found (Figure 2D and 2E). There were 26 (Figure 2D) and 19 (Figure 2E) lncRNAs and only five of the same PCGs in those two sub-modules. A uniquely enriched pathway of all five PCGs (FSTL3, KLF6, MLF1, NR4A3, and SDC4) was “Transcriptional misregulation in cancer” (P = 0.048), suggesting that those five PCGs and their regulating lncRNAs may play roles in transcriptional regulation levels in THCA. It also suggests their potential as biomarkers or therapeutic targets in THCA.

### Diagnostic values of LA16c-380H5.2, RP11-203J24.8, MLF1 and SDC4

**Figure 3:**
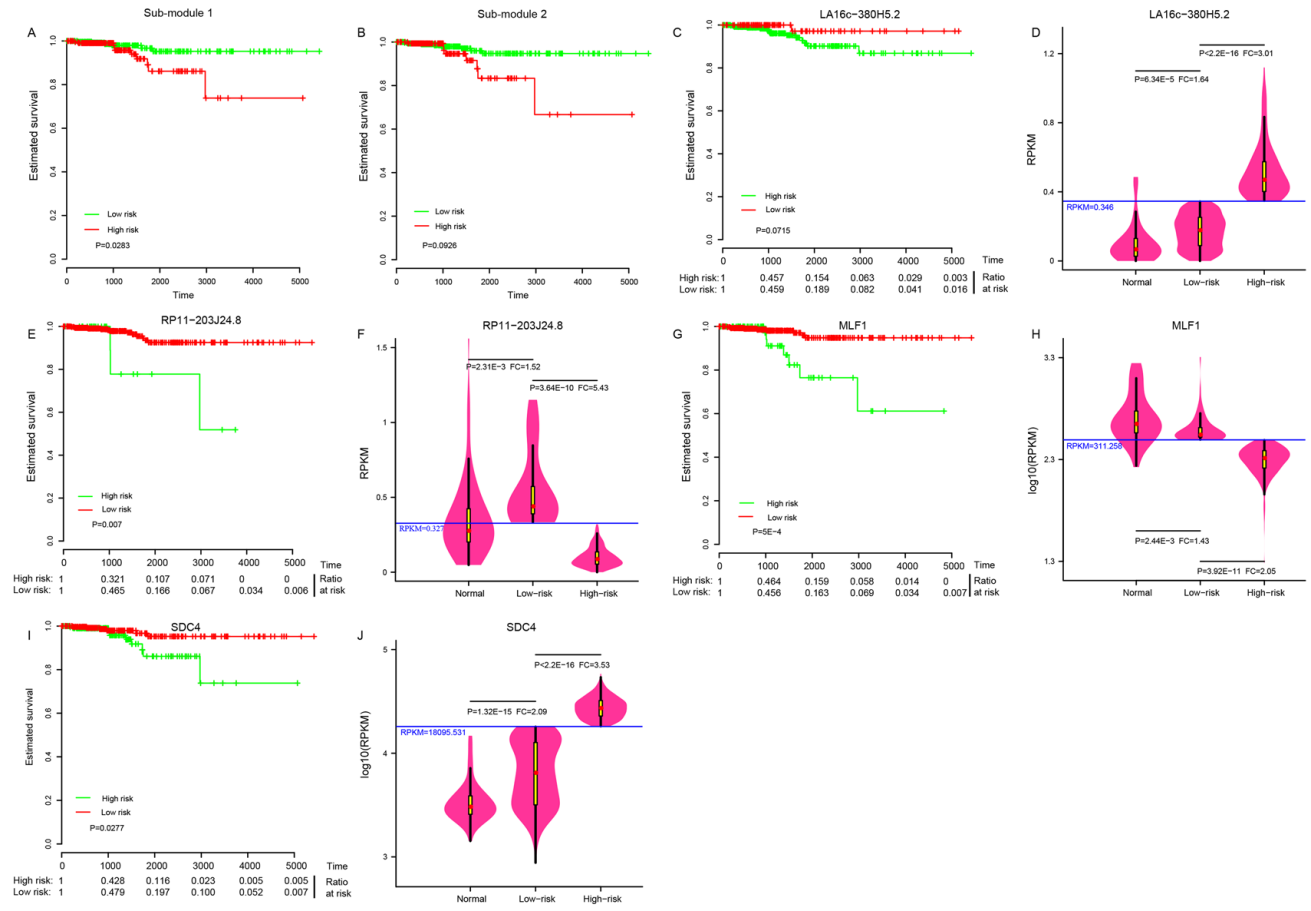
Four potential biomarkers Sub-module 1 **(A)**, instead of sub-module 2 **(B)** could distinguish the high-risk group from the low-risk group in THCA patients. Four elements in sub-module 1: LA16c-380H5.2 **(C)**, RP11-203J24.8 **(E)**, MLF1 **(G)** and SDC4 **(I)** could distinguish the high-risk group from the low-risk group. Comparison of expression levels in the normal, the low-risk and the high-risk groups for LA16c-380H5.2 **(D)**, RP11-203J24.8 **(F)**, MLF1 **(H)** and SDC4 **(J)**.

The 505 patients were classified into either a high-risk group or a low-risk group by sub-module 1 (P = 0.028, Figure 3A), instead of sub-module 2 (P = 0.093, Figure 3B), demonstrating a significant difference in estimated survival time. Just four of the 31 elements in sub-module 1 showed predictive power, and were able to independently distinguish low-risk patients from high-risk individuals at a statistically significant level of 0.1. Those four elements were LA16c-380H5.2 (Figure 3C), RP11-203J24.8 (Figure 3E), MLF1 (Figure 3G), and SDC4 (Figure 3I); the other 27 elements are shown in Supplementary Figure 3. Patients in the high-risk group had a lower survival ratio (Figure 3C, 3E, 3G, 3I) and shorter survival time (Supplementary Table 5) than those in the low-risk group. ROC (receiver operating characteristic) curves were applied to evaluate whether those four elements could distinguish high- or low-risk status. Surprisingly, the AUC (area under the ROC curve) was one with RPKM (reads per kilobase per million mapped) cutoff values of 0.346, 0.327, 311.258, and 18095.531 for LA16c-380H5.2, RP11-203J24.8, MLF1, and SDC4, respectively (Supplementary Figure 4). These RPKM cutoff values could completely differentiate high- or low-risk status with P values less than 1.0E-10 and FC values of more than two (Figure 3D, 3F, 3H and 3J). The expression of LA16c-380H5.2 and SDC4 tended to be up-regulated, while the remaining two (RP11-203J24.8 and MLF1) were down-regulated for patients in the high-risk group, suggesting that their expression levels were closely related to the development of THCA. Interestingly, they all showed obviously different expressions levels in the low risk and normal groups (Figure 3D, 3F, 3H and 3J).

**Figure 4:**
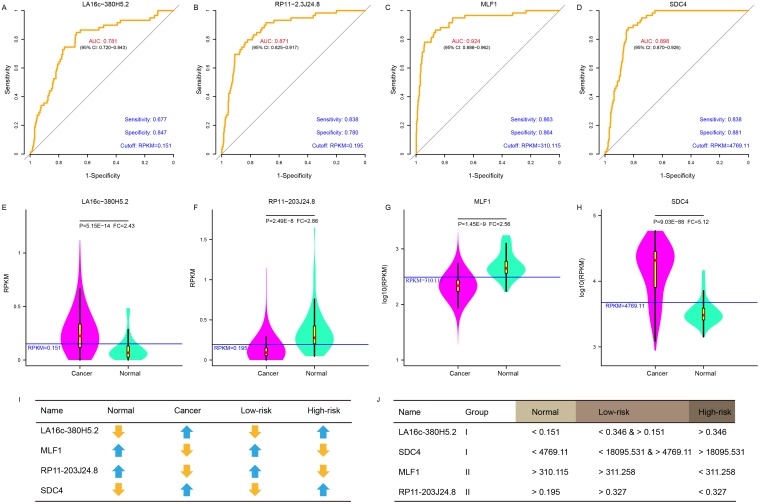
A panel of biomarker candidates with diagnostic values ROC curve of LA16c-380H5.2 **(A)**, RP11-203J24.8 **(B)**, MLF1 **(C)** and SDC4 **(D)** showed high sensitivity and specificity to diagnose THCA patients from normal individuals. Comparison of expression levels in normal and cancer samples for LA16c-380H5.2 **(E)**, RP11-203J24.8 **(F)**, MLF1 **(G)** and SDC4 **(H)**. **(I)** Comparison of expression change trends for four biomarkers in normal and cancer, high-risk and low-risk groups. **(J)** RPKM cutoff values of LA16c-380H5.2, RP11-203J24.8, MLF1 and SDC4 to distinguish the normal, the low-risk or the high-risk groups.

**Figure 5:**
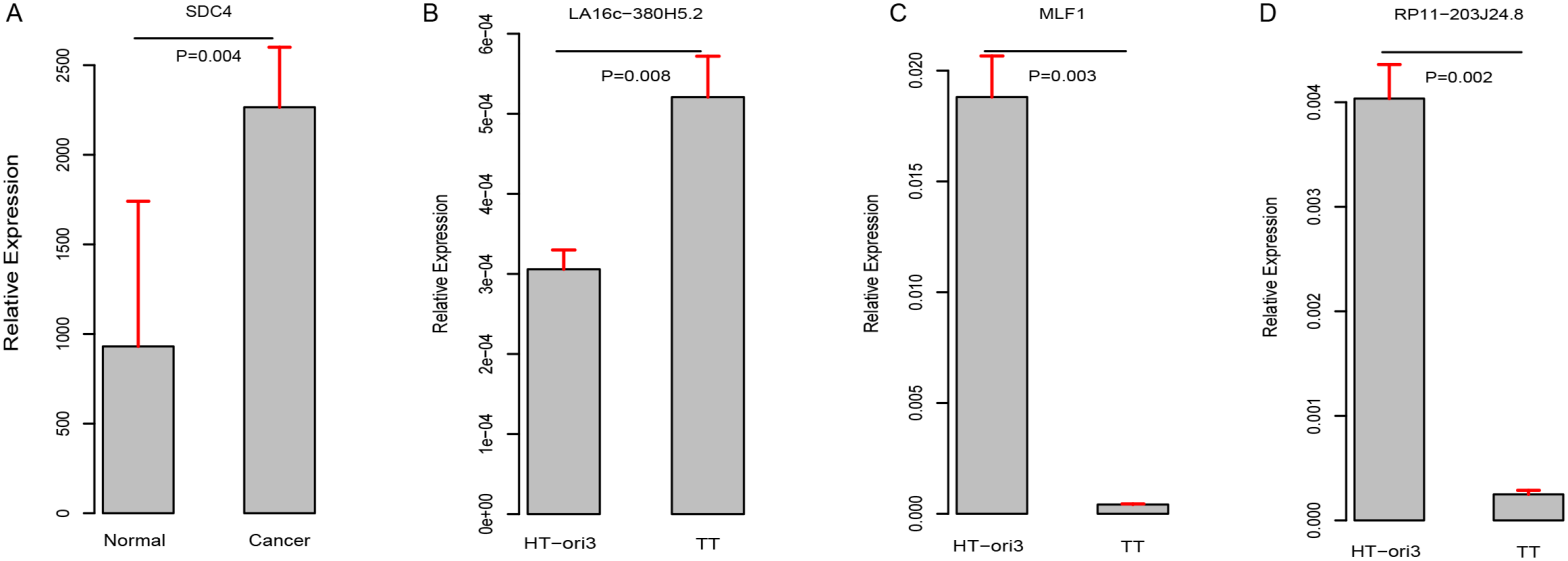
Validation of expression pattern of these four genes **(A–D)**.

ROC curves were further generated to evaluate whether they could diagnose THCA. For LA16c-380H5.2, the AUC reached 0.781 (95% CI: 0.720-0.843) when using the RPKM cutoff value of 0.151, and the sensitivity and specificity were 0.677 and 0.847, respectively (Figure 4A). For RP11-203J24.8, these values are an AUC of 0.871 (95% CI: 0.825-0.917), a RPKM cutoff value of 0.195, and a sensitivity and specificity of 0.838 and 0.780 (Figure 4B). For MLF1, the AUC was 0.924 (95% CI: 0.886-0.962), the RPKM cutoff value was 310.115, and the sensitivity and specificity were 0.863 and 0.864 (Figure 4C). For SDC4, the AUC was 0.898 (95% CI: 0.870-0.926), the RPKM cutoff value was 4769.11, and the sensitivity and specificity were 0.838 and 0.881 (Figure 4D). THCA can be diagnosed accurately using expression levels of these four elements (Figure 4E-4H). These analyses showed that these four elements might serve as outstanding diagnostic biomarkers for THCA, which would be useful in screening biopsies in a histopathologic setting. We confirmed the expression pattern of the SDC4 by other samples (GSE3467 and GSE3678 [43]) from GEO datasets (Figure 5A and Supplementary Figure 5A). And, we also had experimental data about the expression of the remaining three genes between THCA cell lines (TT, B-CPAP and BHT101) and normal thyroid cell lines (HT-ori3) (Figure 5B-5D and Supplementary Figure 5B and 5C).

As shown in (Figure 4E-4H) and (Figure 3D, 3F, 3H, 3J), LA16c-380H5.2 and SDC4 were up-regulated in THCA patients compared to normal samples and also tended to be expressed at higher levels in the high-risk group compared to the low-risk group. In contrast, MLF1 was down-regulated in THCA patients when compared with normal samples and also tended to be expressed at a lower level in the high-risk group compare with the low-risk group. RP11-203J24.8 was also differentially expressed among the normal sample, the low-risk group and the high-risk group. These expression signatures are shown in (Figure 4I), suggesting that these four elements could diagnose THCA patients from normal individuals. Based on the cutoff values of RPKM, they may be divided into two groups with LA16c-380H5.2 and SDC4 in Group I and MLF1 and RP11-203J24.8 in Group II (Figure 4J). Group I could explicitly differentiate the normal, low-risk and high-risk groups. For LA16c-380H5.2, an individual would be diagnosed as normal with an RPKM value less than 0.151, as high-risk with an RPKM value higher than 0.346, and as low-risk with an RPKM value between 0.151 and 0.346. For SDC4, an individual would be considered healthy with an RPKM value less than 4769.11, as high-risk with an RPKM value more than 18095.531, and as low-risk with an RPKM value between 4769.11 and 18095.531. The results of Group II seemed ambiguous, but they may be an accessory to Group I. MLF1, SDC4, LA16c-380H5.2, and RP11-203J24.8 were not in the list of pan-cancer (including THCA) biomarkers reported by *Bogumil Kaczkowski et al.* [44]. Likewise, they were not expressed differentially in a pan-cancer (not including THCA) transcriptome analysis published by *Christopher R Cabanski et al.* [45]. This means that those expression signatures (Figure 4J) could be used to develop a novel diagnostic panel for THCA patients. In comparison, PAX8 (paired box 8), which encodes a member of the paired box family of transcription factors, was involved in thyroid follicular cell development and was thyroid-specifically expressed [46]. TTF1 (thyroid transcription factor 1) was considered to be a thyroid transcription factor that encoded one homeobox protein [47]. They were identified as two potential biomarkers of THCA [48]. In the present study, both PAX8 and TTF1 were expressed at lower levels in THCA patients than in normal samples; however, they were not notable enough to be biomarker candidates, with FC values less than two (Supplementary Figure 2C and 2D).

**Figure 6:**
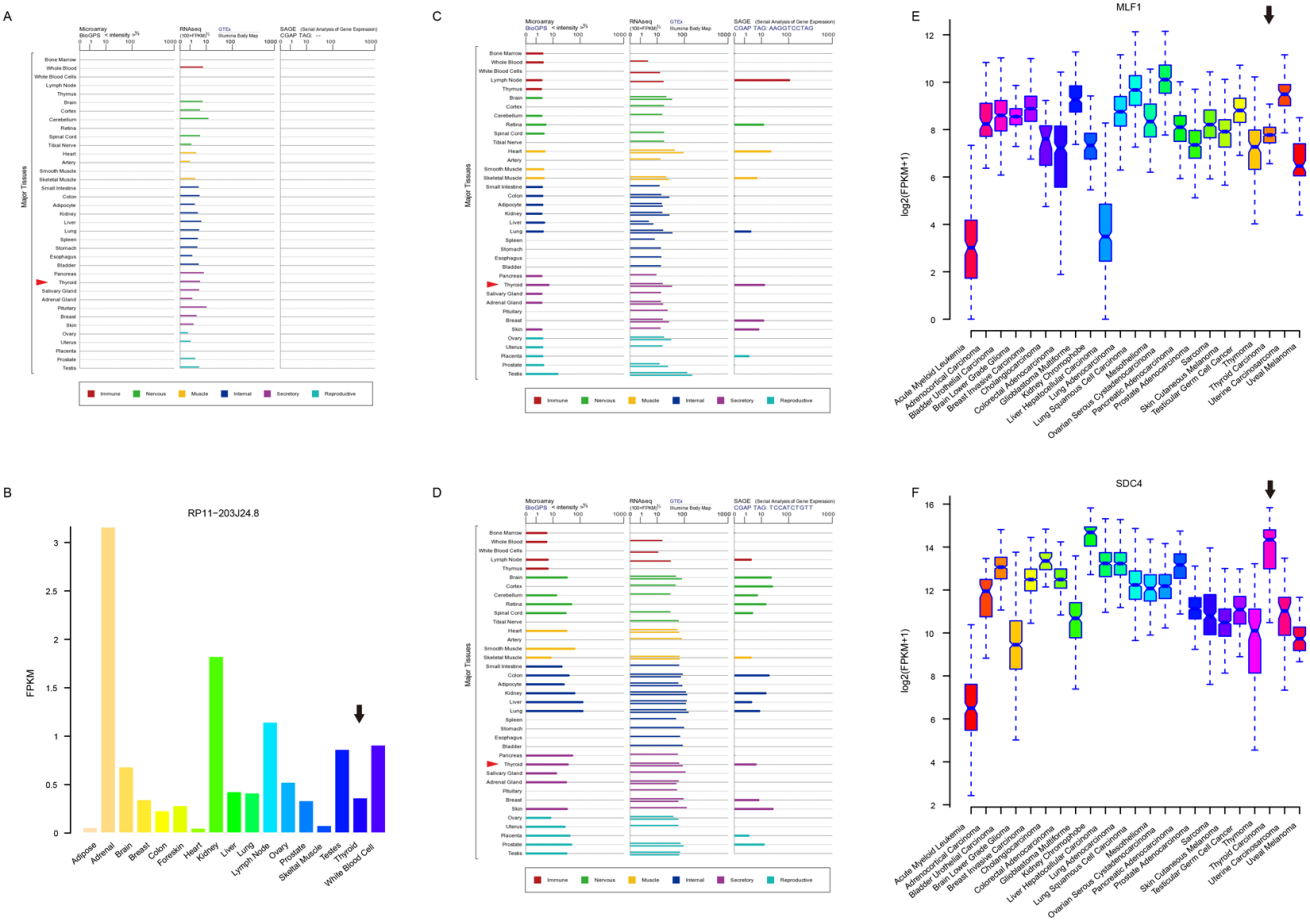
Expression profiles of four biomarker candidates in three public databases Expression profiles of LA16c-380H5.2 **(A)**, MLF1 **(C)** and SDC4 **(D)** in normal tissues from GeneCards database. Expression profiles of RP11-203J24.8 **(B)** in normal tissues from NONCODE database. Expression profiles of MLF1 **(E)** and SDC4 **(F)** in various cancers from cBioPortal database.

To further examine whether these four biomarkers were THCA-specific or thyroid-specific, we retrieved their expression levels from three other independent datasets (Genecards [49], cBioPortal [50], and NONCODE [51]) in FPKM (fragments per kilobase per millions) values. LA16c-380H5.2, which was not found in NONCODE, was found at a low expression level (mean FPKM = 0.16) in the thyroid (Figure 6A). RP11-203J24.8 matched no results in Genecards and cBioPortal, but was expressed in low levels (mean FPKM = 0.365) in the thyroid (Figure 6B). MLF1 was expressed moderately (mean FPKM = 14.44) in the thyroid (Figure 6C). SDC4 was highly expressed (mean FPKM = 75.69) in the thyroid (Figure 6D). All four biomarkers were pervasively expressed in most major human tissues and did not show a thyroid-specific expression pattern. MLF1 was moderately expressed (mean FPKM = 233.73, Figure 6E), while SDC4 was apparently highly expressed (mean FPKM = 19772.64, Figure 6F) in THCA, among various cancers. Although MLF1 and SDC4 were generally highly expressed in multiple types of cancers, their expression levels varied over a large range. Both MLF1 and SDC4 were not THCA-specific features.

### Potential function of LA16c-380H5.2, RP11-203J24.8, MLF1 and SDC4

To explore the functional implication of LA16c-380H5.2 and RP11-203J24.8, both of which were uncharacterized previously in thyroid cells, in THCA tumorigenesis and development, we predicted lncRNA roles by using their targets. Among dysregulation pairs, KLF6 was the unique target of RP11-203J24.8, while the only two targets of LA16c-380H5.2 were, coincidentally, MLF1 and SDC4 (Figure 2B). The duplex structure of these two lncRNAs and their targets was predicted (Supplementary Figure 6A and 6B), and very low MFE (minimum free energy) indicated high accessibility for the sequence. The PCC between KLF6 and RP11-203J24.8 was 0.71 (P = 3.09E-10) in normal samples, but showed a sharp decrease (PCC = 0.188, P = 2.15E-5) in the cancer group (Supplementary Figure 6C). The PCC between MLF1 or SDC4 and LA16c-380H5.2 was 0.779 (P = 3.62E-13) and 0.85 (P = 1.77E-17), respectively, from normal samples and 0.058 (P = 0.194) and 0.35 (P = 5.87E-16) for the cancer group (Supplementary Figure 6D and 6E); both PCC values fell considerably. These results illustrate that these three dysregulation pairs were affiliated with the Type I (Table 2), meaning that LA16c-380H5.2 and RP11-203J24.8 lost some targets in THCA, which may be partly because of the strict criteria of |PCC| > 0.7 and P < 0.001. Therefore, we focused on their targets in normal samples. A total of 147 PCGs were positively or negatively correlated with LA16c-380H5.2 (Supplementary Table 6), and 11 PCGs were positively or negatively correlated with RP11-203J24.8 (Supplementary Table 7). The 147 PCGs were significantly enriched (P < 0.05 and Fold Enrichment > 2) in 67 GO (Gene Ontology) terms and 13 KEGG (Kyoto encyclopedia of genes and genomes) pathways (Supplementary Table 8), including “negative regulation of cell proliferation” (P = 2.41E-4), “negative regulation of cell growth” (P = 0.014), “negative regulation of cell cycle” (P = 0.03), “negative regulation of apoptotic process” (P = 0.009), “activation of MAPK activity” (P = 0.009), and “positive regulation of cell migration” (P = 0.01). Except for those GO functional terms that seemed conflicting in function, some important KEGG pathways that are involved in cancers were also enriched, such as “TNF signaling pathway” (P = 0.003), “p53 signaling pathway” (P = 0.003), “MAPK signaling pathway” (P = 0.028) which was reported by different studies on THCA [2, 4, 17, 28], and “PI3K-Akt signaling pathway” (P = 0.04), showing that the lncRNA LA16c-380H5.2 may promote normal growth and development of thyroid cells. By detecting expression in 505 THCA and 59 normal tissues, we found significantly higher expression of LA16c-380H5.2 in patients (Figure 4E), suggesting that LA16c-380H5.2 may be considered as an oncogene in the thyroid and may be a potential novel therapeutic target for THCA patients. For targets of lncRNA RP11-203J24.8, we found no KEGG pathways enriched, and only one enriched GO term: “intracellular signal transduction” (P = 0.023); its target KLF6 was reported as a cancer driver gene [52] that also showed importance in thyroid normality maintenance.

SDC4 (syndecan 4) encodes a type I transmembrane heparan sulfate proteoglycan that is a main cell adhesion receptor involved in focal adhesion formation, and is required for cell migration [53, 54]. SDC4 has been reported as a cancer driver gene [52] and was up-regulated in our study (FC=5.12, Figure 4H), which was in accordance with two previous studies using microarrays [4, 25]; *Griffith et al.* reported a 3.32 FC [25], and *Chung et al.* detected a 3.90 FC [4]. These results were consistent with the high expression of SDC4 in other malignances (Figure 6F), indicating the oncogene nature of SDC4 in THCA development. MLF1 (myeloid leukemia factor 1) encodes an oncoprotein that plays a role in the phenotypic determination of hemopoietic cells [55]. Translocations between MLF1 and nucleophosmin have been associated with myelodysplastic syndrome (MDS) and acute myeloid leukemia (AML) [56]. Increased MLF1 as an oncogene expression has been correlated with a poor prognosis in AML and with malignant progression in MDS [57, 58]; however, it was significantly down-regulated in THCA (Figure 4G) and expressed at a lower level in the high risk group than in the low risk group (Figure 3H). MLF1’s lowest expression was marvellously in AML among various cancers, and its expression level in THCA was obviously higher than in AML (Figure 6E). Just like SDC4, MLF1 also occupied a critical position in the network and had more interactions than the average gene (Figure 2D), which are common characteristics of cancer driver genes [59, 60]. These results suggested that MLF1 may have an unknown novel function in THCA as a potential cancer driver gene and may maintain the normal cellular growth and development of thyroid, as well as suppressed roles in THCA tumorigenesis.

## DISCUSSION

Successful cancer treatment depends heavily on early detection [44]; however, few biomarkers are routinely used in clinics [61]. Although THCA is a relatively indolent cancer with low mortality [6], it frequently (30-90% of patients) metastasizes in the lymph node [62]. These often predicts poor prognoses in THCA patients in the cervical region [63]. Persistent and recurrent disease rates remain significant [64]. Therefore, it is becoming increasingly important to find reliable and clinically applicable novel biomarkers for diagnosis or prediction.

In THCA, some molecular markers (such as SDC4, PAX8, TTF1, miR-181b and miR-221) have been evaluated to improve preoperative diagnostic or predictive accuracy in patients [25, 46, 47, 65, 66]. For example, *Griffith et al.* reported 39 differentially expressed genes (23 up-regulated and 16 down-regulated, P < 0.05) in 473 samples [25], and *Chung et al.* identified 79 differentially expressed genes (70 up-regulated and 9 down-regulated, FC > 3 and FDR < 0.01) in 26 samples [4]. Among the up-regulated genes, seven genes (MET, TGFA, PROS1, PSD3, SDC4, TUSC3, and P4HA2) were detected in both reports. Only one gene was selected as a biomarker in our study, SDC4 (P=9.03E-88, FC=5.12, Figure 4H), showing that SDC4 is robustly up-regulated in various THCA populations across different studies. While PAX8 and TTF1 might not be exclusive enough to be biomarkers based on our evaluation (Supplementary Figure 2C and 2D).

In addition to genetic factors, increasing evidence has suggested the implication of lncRNAs in the process of cancer occurrence and progression [67, 68]. The lncRNA can significantly inhibit/promote proliferation, migration and apoptosis of a cell, and it is an attractive way to diagnose and cure cancers via the effective control of both cell growth and motility through lncRNAs [10, 69]. However, only a small amount of lncRNAs are known to be involved in THCA pathogenesis. For example, BANCR (BRAF-activated lncRNA) could increase cell proliferation in papillary thyroid cancer (PTC, PTC accounts for approximately 80% of all THCA in adults [70]), and its levels were significantly higher in PTC [18]. PTCSC3 (papillary thyroid carcinoma susceptibility candidate 3) is a tumor suppressor, and was down-regulated in PTC [2, 19, 71]. NAMA (noncoding RNA associated with MAP kinase pathway and growth arrest) was found to be down-regulated in PTC and associated with arresting growth [17]. The expression of PVT1 (LINC00079), which contributes to tumorigenesis through recruiting EZH2 (polycomb enhancer of zeste homolog 2) and regulating TSHR (thyroid-stimulating hormone receptor), was significantly up-regulated in THCA [20]. All these reports show that lncRNA expression abnormality may be one reason of cancer pathogenesis and also suggested its potential as diagnostic biomarkers. In our study, the expression levels of PTCSC3 (P = 9.21E-17, FC = 0.41, Supplementary Figure 2E) and NAMA (P = 5.37E-16, FC = 0.29, Supplementary Figure 2F) in THCA were concordant with those above studies, when compared with the normal samples. BANCR was not present in the GRCh37 reference. PVT1, with slight down-regulation in our study (P = 0.03, FC = 0.746, Supplementary Figure 2G), was not defined as a differentially expressed lncRNA. Which may suggest that different environmental exposures may lead to various changes in diverse populations. This difference may also come from methods (namely, the qRT-PCR method and methods where cancer tissues are compared with adjacent normal tissues) and sample size (84 patients) in its study. Those above analyses about PCG and lncRNA were limited to a small number of samples, but the biomarkers we identified were more believable because of the advantages of a larger sample size and a more advanced platform of NGS. Recently, *Qiuying Li et al* also reported four independent lncRNA biomarkers (RP11-536N17.1, RP11-508M8.1, AC026150.8 and CTD-2139B15.2), which were different from the four biomarkers identified in our study, associated with prognosis from the same TCGA-THCA dataset [72]. There are many difference between those two researches. *Qiuying Li et al* randomly divided THCA patients into two distinct sets of equal size: the 246-patient training dataset and the 246-patient testing dataset, they studied lncRNAs from the GENCODE Resource (version 19) and used TCGA RNA-sequencing data in the BAM file, and differentially expressed lncRNAs were identified using a paired student t-test, their prognostic biomarkers were focused on the survival and recurrence prediction. While we used the THCA patients as a whole, we focused on cancer PCGs and their regulating lncRNAs from the GRCH37 human genome assembly and used TCGA RNA-sequencing data in the FASTQ format, and differentially expressed PCGs and lncRNAs were identified using Fisher's exact test or student's t test, biomarkers we identified were focused on their diagnostic values.

Furthermore, the number of DNA mutations is highly heterogeneous among various types of cancers [73]. *Eduard Porta-Pardo et al.* previously reported that THCA had only 11 missense mutations per sample (4420 mutations in 401 samples), which was the lowest in 23 types of cancer (5989 samples) from TCGA, while melanoma had the highest number, with 429 missense mutations per sample [52]. Since the relatively small number of DNA mutations in THCA patients may restrict the consideration of them as biomarkers, aberrantly expressed PCGs and lncRNAs of cancers could be a good alternative. Herein, we demonstrated the utility of a novel panel of diagnostic biomarker candidates including two PCGs (MLF1 and SDC4) and two lncRNAs (LA16c-380H5.2 and RP11-203J24.8) from the dysregulation network based on expression signatures (Figure 4J). Although the number of samples used for creating this panel was limited, the strict criteria used for selecting them provides a strong signature for biomarker validation. Fine needle aspiration, which is considered a gold standard for differential diagnosis, has a diagnostic sensitivity of 0.83-0.98 and specificity of 0.70-0.92 in THCA [74, 75]. Here, we obtained a satisfying result with a sensitivity of 0.677-0.863 and specificity of 0.780-0.881 (Figure 4A-4D). The improved ability to detect RNA, especially by *in situ* hybridization, RT-PCR, and sequencing techniques, will make RNA easily accessible to clinical applications and will improve the diagnosis or prognosis of THCA.

However, there are some limitations that should be acknowledged in our study. It should be emphasized that these four identified potential biomarkers were predicted by bioinformatic methods, and they were just validated in cell lines. Although substantial computational evidence for the diagnostic significance had been revealed, the underlying mechanisms of these four biomarkers in the development of THCA are still unclear. Both tumors and normal tissues are complex mixtures that include multiple types of cells, such as cancer cells, infiltrating lymphocytes and blood vessels, and variations in gene expression may thus simply reflect differences in cell composition [44]. Sample heterogeneity with respect to types of THCA, clinical activity and severity might impair the analysis. Random changes that exist in patients may be another confounding factor; for example, GAPDH is a house-keeping gene and is often used in *in vivo disc* research [76]; however, its expression levels changed markedly in both the THCA group and the normal group (Supplementary Figure 2H). The catalogue of cancer biomarkers is far from complete, and it is difficult to extend it by simply increasing the size of samples [77]. An alternative approach towards that goal is to integrate various types of biological knowledge to increase the statistical power of the analysis [52]. Moreover, because of extreme complexity and individual diversity, the current gene-centric paradigm in cancer biology may not be enough to explain the complex genotype-phenotype relationships [78–80]. It is expected that the information of DNA and epigenetic variation and protein-protein interaction profiles of cancer patients will be combined with our RNA-centric analysis.

## MATERIALS AND METHODS

### Sequencing data analysis

The results shown here are in whole based upon data generated by the TCGA Research Network: http://cancergenome.nih.gov/. The downloaded RNA sequencing data used in this study are in FASTQ format, including 505 patients and 59 healthy individuals; those 564 sample IDs are provided in Supplementary Table 1. Bowtie software [81] with the default parameters was used to align RNA sequencing reads (48 nucleotides length) to the GRCH37 human genome assembly downloaded from the Ensembl database (http://asia.ensembl.org). These uniquely mapped reads in the genome were used to identify unambiguous transcription. Reads aligned to more than one locus were discarded. The expression level of transcripts was quantified by calculating the RPKM (reads per kilobase per million mapped) value. Genes were categorized as “protein coding” and “non-coding” based on an Ensembl annotation file in the GTF format. Among non-coding genes, rRNAs, tRNAs, miRNAs, snoRNAs and other known classes of RNAs were excluded, and lncRNAs were defined as all non-coding genes longer than 200 nucleotides and not belonging to other RNA categories. There were 18078 protein coding genes (PCGs) and 12727 lncRNA in the reference.

### Differential expression analysis of cancer and normal samples

To identify the difference in PCG or lncRNA expression in cancer samples versus normal samples, we used the Wilcoxon rank sum test. For each feature (lncRNA or PCG), the read count was used as the input expression datum; we then calculated the frequency of expression in cancer and normal samples. If the ratio of the zero-value for the expression level (not detected, read count = 0) was more than 30% in cancer or in normal samples, a two-sided Fisher’s exact test with an adjustment for multiple testing by the *Benjamini-Hochberg* method was used, with the thresholds of FDR < 0.01 and |FC| (absolute value of the fold change) > 2. Otherwise, Student's t test with the same adjustment was applied, with FDR < 0.05 and |FC| > 2. The FC was calculated by taking the higher mean RPKM value divided by the lower mean RPKM value in the THCA or normal samples. Unsupervised hierarchical clustering was done by R software (version 3.3.2, http://www.r-project.org/).

Expression profiles of SDC4 and MLF1 in various cancers were queried in the cBioPortal (http://www.cbioportal.org) [50]. LA16c-380H5.2, SDC4, and MLF1 expression values in major normal tissues were downloaded from the GeneCards (http://www.genecards.org) [49]. RP11-203J24.8 expression profiles in major normal tissues were retrieved from the NONCODE (http://www.noncode.org) [51].

### Construction of the regulatory network

Pearson’s correlation coefficient (PCC) was calculated by in-house R- scripts, and was utilized to evaluate the co-expression relationship between lncRNA and PCG. Co-expressed pairs were defined with a cutoff of |PCC| ≥ 0.7 and P < 0.001. Network interactions were graphed using Cytoscape software (version 3.2.1) [82]. Two sub-modules were identified by CytoCluster [83], with the HC-PIN clustering algorithm and other parameters as default, which is a plugin in Cytoscape.

### Data visualization

The Kaplan-Meier survival curves of over-all survival was obtained using the Survival package [84]. The receiver operating characteristic (ROC) and the area under the ROC curves (AUC) values were obtained from the pROC package [85]. Unless otherwise specified, data were analyzed and visualized using R software (version 3.3.2).

### Duplex structure prediction

The duplex structure was predicted by RNAplex and RNAduplex in the ViennaRNA package [86], using the DNA sequence of the PCG with 2000bp upstream and downstream and the RNA sequence of the lncRNA, which were retrieved from the GRCH37 human genome reference. The range of the predicted duplex structure in the two sequences is in the format “from, to : from, to”. The minimum free energy (MFE) is in kcal/mol. The predicted structure is in the dot-bracket format with a “&” separating the two sequences. A dot in the format represents an unpaired position, while a base pair (i, j) is represented by a pair of matching parentheses at positions i and j.

### Pathway analysis

For enrichment analysis to explore their biological effects, PCGs were submitted to the DAVID (http://david.abcc.ncifcrf.gov/) [87]. The reporting of the enriched results was limited to Gene Ontology (GO) terms and Kyoto encyclopedia of genes and genomes (KEGG) pathway categories using the functional annotation clustering and functional annotation chart options. The GO terms and KEGG pathways with P values of < 0.05 were considered as significantly enriched function annotations.

### Reagents and cell culture

F12K medium (Life, Cat. 21127022), RPMI 1640 medium (Gibco, Cat. 11875093), DMEM(H) (Gibco, Cat. 11965092), NEAA (Invitrogen, Cat. 11140050), Glutamax (Invitrogen, Cat. 35050061), Sodium Pyruvate (Invitrogen, Cat. 11360070), Fetal bovine serum (Gibco, Cat. 10099141), penicillin/streptomycin solution (P/S, Cat. 0503), TRIzol™ Reagent (Invitrogen, Cat. 15596018), reverse transcription kit (Takara Bio Inc, Liaoning, China, Cat. RR036B (A × 4)), SYBR Premix Ex Taq kit (Takara Bio Inc, Liaoning, China, Cat. RR420B).

The human thyroid medullary carcinoma cell line (TT), human papillary thyroid carcinoma cell lines (B-CPAP and BHT101) and human normal thyroid cell line (HT-ori3) were used in the current study. Those cell lines were purchased from Stem Cell Bank, Chinese Academy of Sciences (Shanghai, China). TT cells were cultured with F12K medium containing 10% (vol/vol) FBS and 1% (vol/vol) P/S at 37°C with 5% CO_2_. B-CPAP cells were cultured with RPMI 1640 medium containing 10% (vol/vol) FBS, 1% (vol/vol) P/S, 1% NEAA, 1% Glutamax and 1% Sodium Pyruvate at 37°C with 5% CO_2_. BHT101 cells were cultured with DMEM(H) medium containing 20% (vol/vol) FBS and 1% (vol/vol) P/S. HT-ori3 cells were cultured with 1640 medium containing 10% (vol/vol) FBS and 1% (vol/vol) P/S.

### RNA isolation

TT cells, B-CPAP cells, BHT101 cells, and HT-ori3 cells were cultured in the specific medium and harvested at 80% density. The cell pellets were dissolved in 1 ml Trizol solution and isolated according to a stand RNA isolation procedure. The RNA concentration in each sample was quantified using a spectrophotometer at 260 nm, the purity of RNA was assessed by measuring OD260/OD280 ratio (range 1.85–2.00).

### Real-time PCR

Total RNA of different cell lines was extracted, and then the cDNA were synthesized with reverse transcription. Real-time PCR reactions were carried out in a final volume of 25 ul, using SYBR Premix ExTaq kit, 0.4 mM of each primer, and 200 ng of cDNA template. Each individual sample was run in triplicate wells. PCR amplification cycles were performed using iQ™5 Multicolor Real-Time PCR Detection System (Bio-RAD). The reactions were initially denatured at 95°C for 3 min, followed by 50 cycles of 95°C for 10s, 55°C for 15s, 72°C for 35s. The expression levels were calculated using the 2^-ΔΔCt^ method and normalized to β-actin. The sequences of oligonucleotide primers were showed in Supplementary Table 9, primers were designed using NCBI primer-blast (http://www.ncbi.nlm.nih.gov/tools/primer-blast/). Experimental data were presented as the mean ± SD. Statistical significance between the groups was calculated by a two-tailed Student’s t test.

## SUPPLEMENTARY MATERIALS FIGURES AND TABLES











## Abbreviations

AML: acute myeloid leukemia
AUC: area under the ROC curves
BANCR: BRAF-activated lncRNA
EZH2: polycomb enhancer of zeste homolog 2
FC: fold change
FPKM: fragments per kilobase per millions
GO: Gene Ontology
KEGG: Kyoto encyclopedia of genes and genomes
lncRNA: long non-coding RNA
MDS: myelodysplastic syndrome
MFE: minimum free energy
MLF1: myeloid leukemia factor 1
NAMA: noncoding RNA associated with MAP kinase pathway and growth arrest
NGS: the next generation sequencing
PAX8: paired box 8
PCC: pearson correlation coefficient
PCG: protein coding gene
PTC: papillary thyroid cancer
PTCSC3: papillary thyroid carcinoma susceptibility candidate 3
ROC: receiver operating characteristic
RPKM: the reads per kilobase per million mapped
SDC4: syndecan 4
TCGA: The Cancer Genome Atlas
THCA: thyroid carcinoma
TSHR: thyroid-stimulating hormone receptor
TTF1: thyroid transcription factor 1
