# Supplementary material for: Identification of novel diagnostic biomarkers for thyroid carcinoma
Source: Oncotarget. 2017 Dec 4;8(67):111551–66. doi: 10.18632/oncotarget.22873 (PMC5762342; doi:10.18632/oncotarget.22873)
Supplement: Supplementary file 1 [file oncotarget-08-111551-s001.pdf]

Identification of novel diagnostic biomarkers for thyroid carcinoma

SUPPLEMENTARY MATERIALS

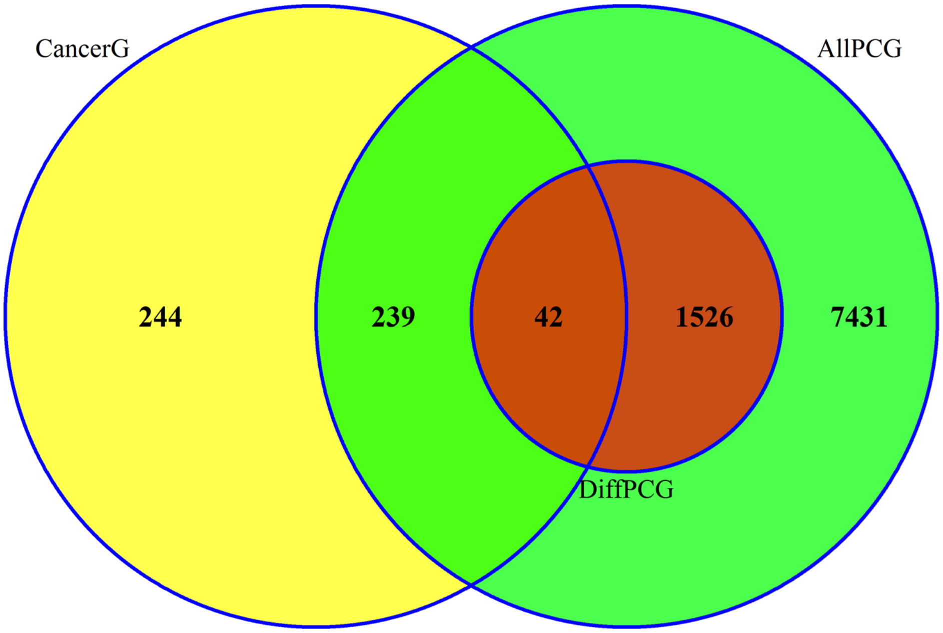

Supplementary Figure 1: The number of "AllPCG", "DiffPCG", and "CancerG". AllPCG": all PCGs in the Ensembl reference which were co-expressed with the differentially expressed lncRNAs, "DiffPCG": differentially expressed PCGs, "CancerG": differentially expressed PCGs that were also cancer genes.

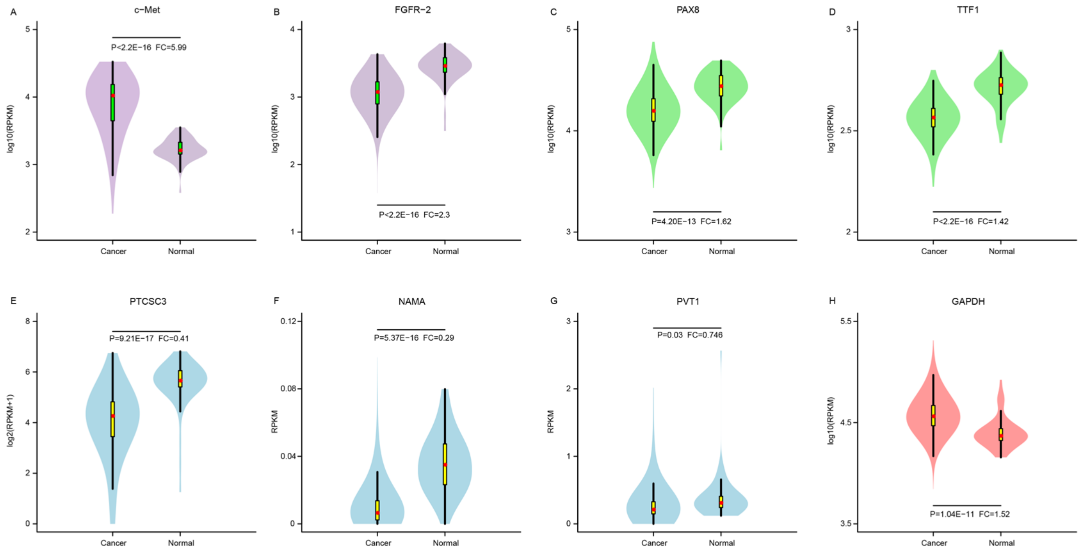

Supplementary Figure 2: Expression comparison of c-Met **(A)**, FGFR-2 **(B)**, PAX8 **(C)**, TTF1 (D), PTCSC3 **(E)**, NAMA **(F)**, PVT1 **(G)** and GAPDH **(H)** between THCA and normal samples.

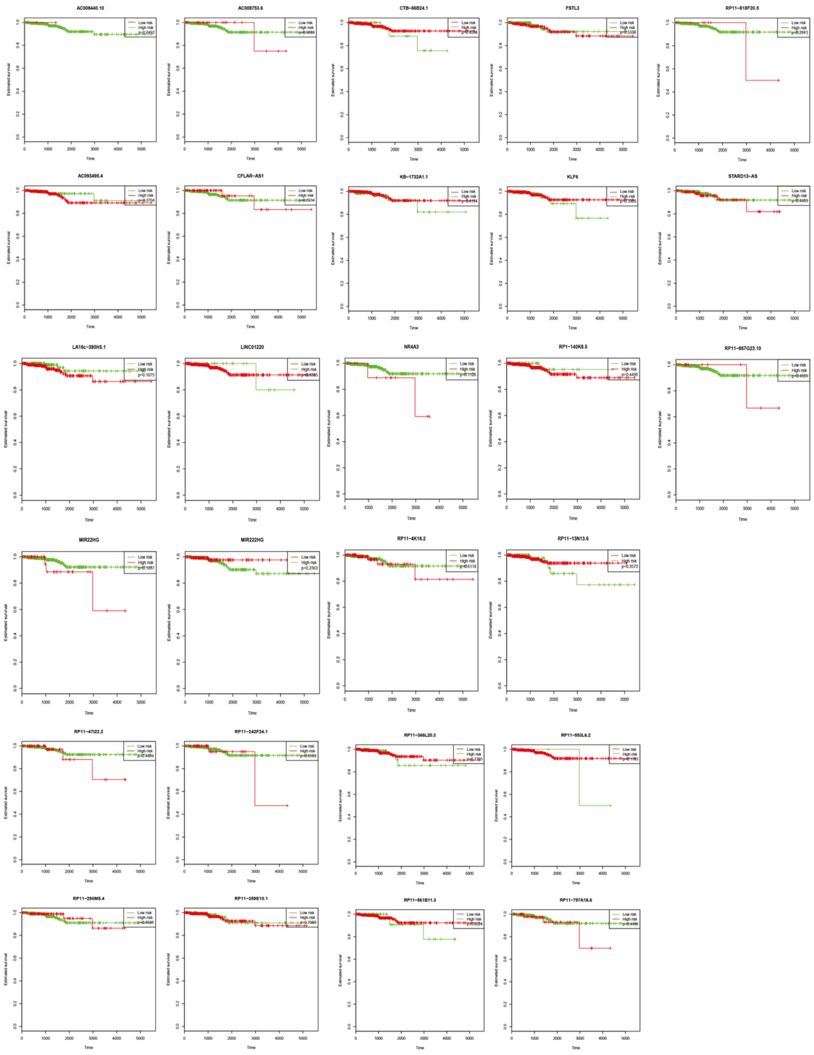

Supplementary Figure 3: Survival curve of 17 elements in sub-module 1.

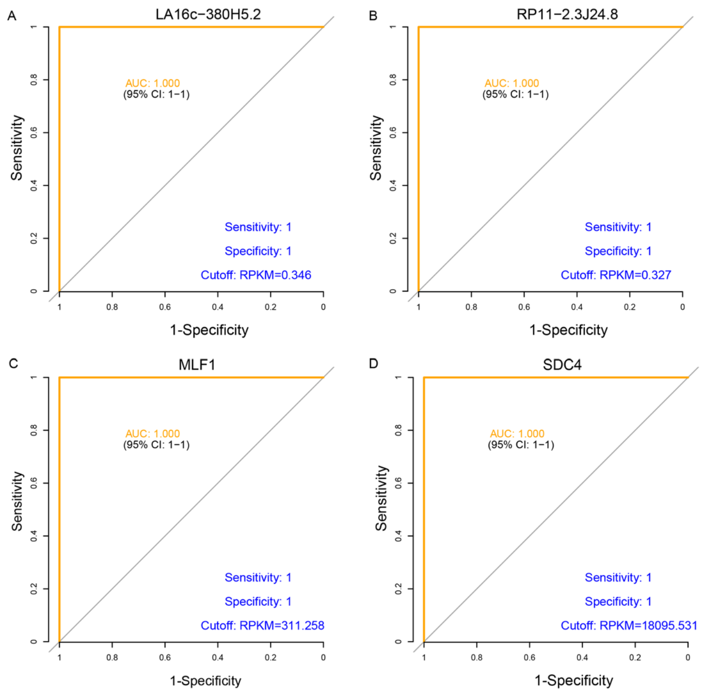

Supplementary Figure 4: ROC curve of four potential biomarkers to distinguish the low-risk group from the high-risk group in THCA population.

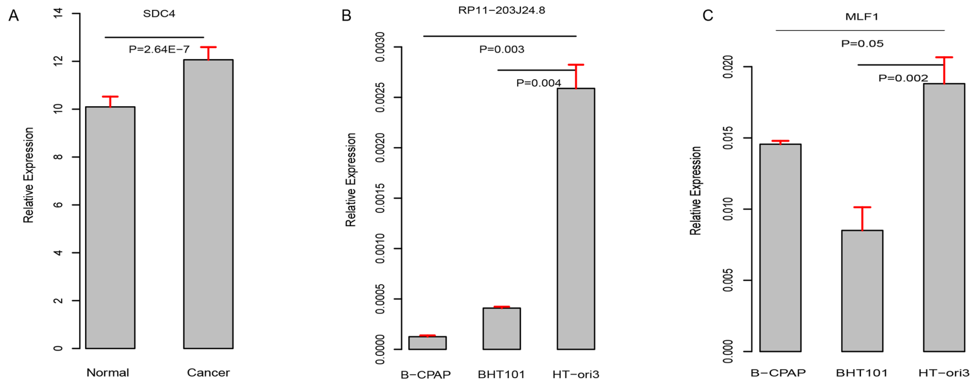

Supplementary Figure 5: Validation of expression pattern of SDC **(A)**, RP11-203J24.8 **(B)** and MLF1 **(C)**.

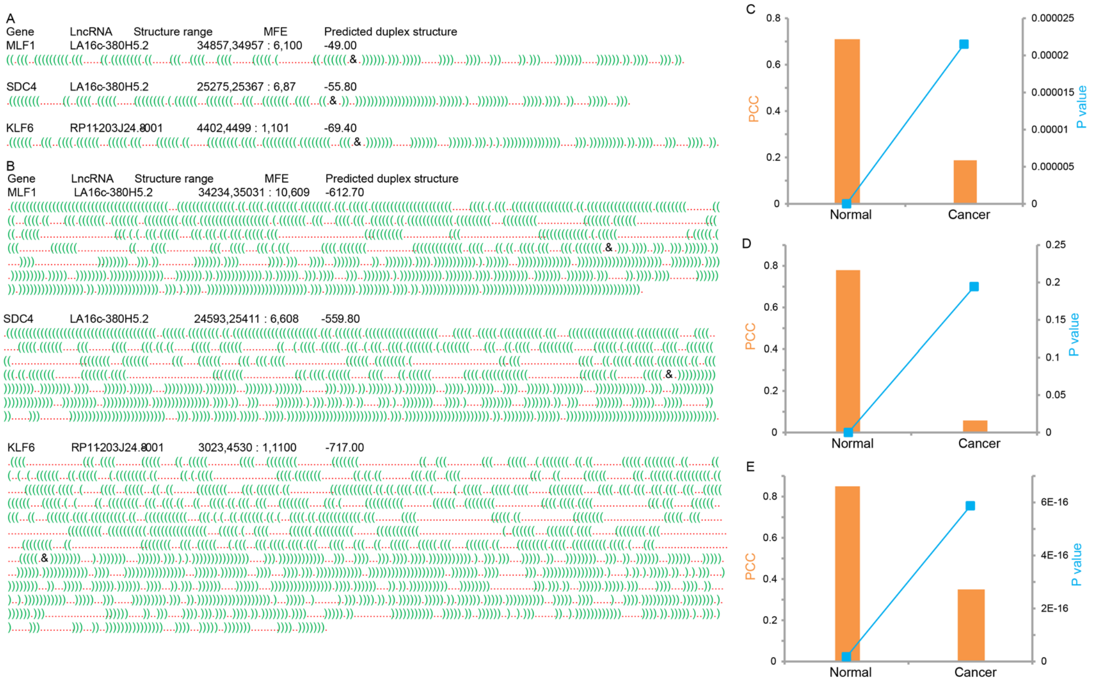

Supplementary Figure 6: Potential interactions among four biomarkers. The duplex structure was predicted by RNAplex **(A)** and RNAduplex **(B)** for lncRNAs and their target PCGs. The range of the predicted duplex structure in the two sequences is in the format "from, to : from, to". The minimum free energy (MFE) is in kcal/mol. The predicted structure is in the dot-bracket format with a "&" separating the two sequences. A dot in the format represents an unpaired position, while a base pair (i, j) is represented by a pair of matching parentheses at positions i and j. Representation of PCC between RP11-203J24.8 and KLF6 **(C)**, between LA16C-380H5.2 and MLF1 **(D)**, between LA16C-380H5.2 and SDC4 **(E)**.

Supplementary Table 1: Sample list from TCGA

See Supplementary File 1

Supplementary Table 2: List of differentially expressed PCGs and lncRNAs between THCA and normal samples

See Supplementary File 2

Supplementary Table 3: Results of KEGG pathway enrichment analysis using differentially expressed PCGs

Catalog	Term	Count	P Value	FE
hsa04080	Neuroactive ligand-receptor interaction	91	1.38E-15	2.33
hsa04512	ECM-receptor interaction	34	2.56E-08	2.77
hsa05033	Nicotine addiction	20	4.06E-07	3.54
hsa04060	Cytokine-cytokine receptor interaction	58	8.54E-06	1.79
hsa04020	Calcium signaling pathway	47	2.38E-05	1.86
hsa04360	Axon guidance	35	1.14E-04	1.96
hsa04151	PI3K-Akt signaling pathway	74	1.63E-04	1.52
hsa05144	Malaria	18	2.29E-04	2.6
hsa04610	Complement and coagulation cascades	22	3.58E-04	2.26
hsa00350	Tyrosine metabolism	14	5.87E-04	2.83
hsa04510	Focal adhesion	47	8.04E-04	1.62
hsa04514	Cell adhesion molecules (CAMs)	35	0.0011	1.75
hsa05146	Amoebiasis	28	0.0013	1.87
hsa05412	Arrhythmogenic right ventricular cardiomyopathy (ARVC)	21	0.0014	2.09
hsa04974	Protein digestion and absorption	24	0.002	1.93
hsa04024	cAMP signaling pathway	44	0.0021	1.57
hsa00830	Retinol metabolism	19	0.003	2.07
hsa00565	Ether lipid metabolism	14	0.0075	2.2
hsa05031	Amphetamine addiction	18	0.0086	1.93
hsa02010	ABC transporters	13	0.0161	2.09
hsa04726	Serotonergic synapse	25	0.0196	1.59
hsa03320	PPAR signaling pathway	17	0.0216	1.8
hsa05410	Hypertrophic cardiomyopathy (HCM)	19	0.0217	1.72
hsa00601	Glycosphingolipid biosynthesis - lacto and neolacto series	9	0.0228	2.45
hsa05414	Dilated cardiomyopathy	20	0.0228	1.69
hsa00982	Drug metabolism - cytochrome P450	17	0.0247	1.77
hsa04727	GABAergic synapse	20	0.0256	1.67
hsa00980	Metabolism of xenobiotics by cytochrome P450	18	0.0263	1.72
hsa05032	Morphine addiction	21	0.0266	1.63
hsa05200	Pathways in cancer	70	0.0281	1.26
hsa00340	Histidine metabolism	8	0.0343	2.46
hsa00500	Starch and sucrose metabolism	10	0.0348	2.15
hsa05030	Cocaine addiction	13	0.0362	1.88
hsa04640	Hematopoietic cell lineage	19	0.0476	1.58

Supplementary Table 4: List of the 42 differentially expressed PCGs that were also cancer PCGs

ENSG00000213190
ENSG00000005073
ENSG00000105976
ENSG00000106031
ENSG00000108821
ENSG00000108924
ENSG00000113263
ENSG00000119866
ENSG00000123364
ENSG00000167751
ENSG00000175832
ENSG00000126752
ENSG00000127946
ENSG00000152217
ENSG00000157765
ENSG00000127083
ENSG00000127152
ENSG00000142611
ENSG00000147889
ENSG00000157404
ENSG00000164398
ENSG00000171791
ENSG00000184937
ENSG00000066468
ENSG00000168685
ENSG00000185499
ENSG00000134574
ENSG00000140937
ENSG00000147257
ENSG00000067082
ENSG00000070371
ENSG00000070404
ENSG00000079102
ENSG00000109906
ENSG00000113594
ENSG00000117400
ENSG00000119508
ENSG00000124145
ENSG00000175643
ENSG00000213190
ENSG00000149948
ENSG00000157388
ENSG00000178053

Supplementary Table 5: The comparison of survival time of THCA patients in low risk and high risk

Ensembl ID	Gene symbol	Chromosomal position	Survival time in high risk (Day)	Survival time in low risk (Day)
ENSG00000178053	MLF1	Chr. 3: 158,571,163- 158,607,252 (+)	1196	1221
ENSG00000124145	SDC4	Chr. 20: 45,325,288- 45,348,424 (-)	1075	1323
ENSG00000262362	LA16c-380H5.2	Chr. 16: 3,003,431- 3,005,101 (-)	1187	1313
ENSG00000227218	RP11-203J24.8	Chr. 9: 127,934,503- 127,940,952 (+)	1044	1228

Supplementary Table 6: Targets of lncRNA LA16c-380H5.2 in normal samples

See Supplementary File 3

Supplementary Table 7: Targets of lncRNA RP11-203J24.8 in normal samples

ID	Change in THCA
ENSG00000067082	Down-regulated
ENSG00000100784	Down-regulated
ENSG00000102098	Down-regulated
ENSG00000109046	
ENSG00000116285	
ENSG00000118523	Down-regulated
ENSG00000126003	
ENSG00000151164	
ENSG00000156313	
ENSG00000171456	
ENSG00000213066	

Supplementary Table 8: GO and KEGG enrichment analysis for targets of lncRNA LA16c-380H5.2 in normal samples

See Supplementary File 4

Supplementary Table 9: The sequences of oligonucleotide primers

LA16c-380H5.2	
Forward primer	GGGGAAAATGGAAGACCCGAA
Reverse primer	GGGGGTTAGAGCCCAGTTTG
RP11-203J24.8	
Forward primer	CAGAATCGGCCTTCAGGGTT
Reverse primer	CCCGTTTCTGACCATCCCAA
SDC4	
Forward Primer	GGACCTCCTAGAAGGCCGATA
Reverse Primer	AGGGCCGATCATGGAGTCTT
MLF1	
Forward Primer	CAAACCAGGACGACACAATCT
Reverse Primer	ATGGCTGGACTTTGTTGAGGT
actin	
Forward primer	CATGTACGTTGCTATCCAGGC
Reverse primer	CTCCTTAATGTCACGCACGAT
